# Emotion regulation strategies and the two-dimensional model of adult attachment: a pilot study

**DOI:** 10.3389/fnbeh.2023.1141607

**Published:** 2023-07-07

**Authors:** Marcos Domic-Siede, Mónica Guzmán-González, Josefa Burgos, Catalina Carvallo, Camila Flores-Guerra, Constanza Fredes-Valenzuela, Javiera Suazo, Oscar Véliz-García, Carlos Calderón, Andrea Sánchez-Corzo, Marcela Perrone-Bertolotti, Jennifer Marín-Medina

**Affiliations:** ^1^Núcleo de Investigación en Neurociencia Cognitiva y Afectiva, Laboratorio de Neurociencia Cognitiva, Escuela de Psicología, Universidad Católica del Norte, Antofagasta, Chile; ^2^Laboratorio de Neurociencia Cognitiva, Escuela de Psicología, Universidad Católica del Norte, Antofagasta, Chile; ^3^Multimodal Functional Brain Imaging and Neurorehabilitation Hub, Department of Diagnostic Imaging, St. Jude Children’s Research Hospital, Memphis, TN, United States; ^4^Université Grenoble Alpes, Université Savoie Mont Blanc, CNRS, LPNC, Grenoble, France; ^5^Institut Universitaire de France, Paris, France; ^6^Unidad de Terapia Familiar, Centro de Intervención y Asesoría Psicosocial CIAP, Escuela de Psicología, Universidad Católica del Norte, Antofagasta, Chile

**Keywords:** emotion regulation, cognitive reappraisal, expressive suppression, adult attachment, ECR-12

## Abstract

**Introduction:**

Emotion Regulation plays a crucial role in human’s daily lives. Extensive research has shown that people with different attachment orientations exhibit divergencies in how they perform emotion regulation strategies.

**Methods:**

44 adults performed an experimental emotion regulation task in which they were instructed to attend, reappraise, or suppress their emotions while viewing negative and neutral images taken from *the International Affective Picture System* (IAPS). Afterward, participants rated valence, arousal, and emotional dominance elicited by the images. Additionally, attachment orientations were measured using the ECR-12 questionnaire.

**Results:**

Results showed a relationship between attachment avoidance and the level of arousal during the reappraisal condition; specifically, the higher attachment avoidance levels, the greater the emotional intensity during the implementation of cognitive reappraisal strategy. Such results suggest an association between failing in downregulate intense emotions using cognitive reappraisal when there are higher levels of attachment avoidance. Consistently, we also found that lower dominance during reappraisal was associated with more levels of avoidance.

**Conclusion:**

These results indicate that people with higher levels of attachment avoidance experience difficulties when using the cognitive reappraisal strategy to reduce the emotional impact produced by negative emotional stimuli. Our findings reinforce the idea that avoidant people experience high physiological activation when experience emotions.

## 1. Introduction

Emotion regulation is the ability to exert control or modulate emotional states (Gross, 1998). Emotional regulation affects the subjective experience, the physiological activation intensity, as well as the expression of emotions, and also have a great impact on people’s health (Gross and Thompson, 2007; Koole, 2009; Kring and Sloan, 2009). Attachment dimension or orientations, consist of organized patterns of cognitions, emotions, and behaviors resulting from the repeated interactions with significant attachment figures (Fraley and Shaver, 2000). Broad evidence indicates that emotional regulation and attachment orientations are closely related and may affect mental (Gresham and Gullone, 2012; Mortazavizadeh and Forstmeier, 2018; Mikulincer and Shaver, 2019) and even physical health (Schmidt et al., 2002).

Attachment anxiety and attachment avoidance are two dimensions that are used to describe romantic attachment and how it relates to the model of self and others, respectively (Brennan et al., 1998; Mikulincer and Shaver, 2016). On one hand, attachment anxiety is characterized by a fear of being abandoned in relationships and a negative view of the self. Those with high levels of attachment anxiety often display a strong need for approval, exaggerated protest reactions, and a constant search for emotional reassurance and closeness in their relationships. On the other hand, attachment avoidance is portrayed by discomfort with intimacy and dependence, a reluctance to seek support, and a tendency to inhibit emotional needs. This avoidance is often driven by the belief that others will reject them due to a negative model of others (Shaver and Mikulincer, 2002; Mikulincer et al., 2003). Individuals who have higher levels of attachment anxiety and/or higher attachment avoidance are typically considered to have a more insecurely attached (Guzmán-González et al., 2020).

Attachment representations and close relationships are related concepts, but they refer to different aspects of our social and emotional lives. Attachment representations refer to the mental models we have of how close relationships work. These models are based on our early experiences with caregivers and shape our expectations, beliefs, and behaviors in future relationships. Attachment representations can be either secure or insecure, with the latter being further divided into different types of insecure attachment (e.g., avoidant, ambivalent, disorganized). Attachment representations are often assessed through measures like the Adult Attachment Interview (AAI). Close relationships, on the other hand, refer to the actual bonds we form with other people. These can be romantic relationships, friendships, or other types of close connections. Close relationships involve a sense of emotional intimacy, mutual support, and shared experiences. Attachment theory proposes that the quality of early attachment experiences with primary caregivers shapes the nature of later intimate relationships. Close relationships can provide a valuable context for assessing attachment patterns, as they often involve strong emotional bonds and interdependence. Attachment patterns can be observed through behaviors such as seeking proximity and support from a partner, expressing vulnerability and emotional needs, and responding to separation or distress with either seeking comfort or withdrawing. Therefore, close relationships can be useful for evaluating attachment patterns, commonly two dimensions of attachment (anxiety and avoidance) and identifying potential areas for growth and improvement (Brennan et al., 1998; Del Giudice and Belsky, 2010; Van IJzendoorn and Bakermans-Kranenburg, 2010; Cassidy and Shaver, 2016; Mikulincer and Shaver, 2016; Guzmán-González et al., 2020). Our study conceptualizes the construct of attachment as dimensions or orientations.

Given that various investigations have shown that emotional regulation difficulties and high levels of attachment insecurity underlie various health problems (Hu et al., 2014), it is important to conduct research in the areas of emotion regulation and attachment. Moreover, maladaptive strategies in managing emotional arousal can negatively affect cognitive processes and behavior which can lead to the development of psychopathology (Vatan and Pellitteri, 2016). For example, a deficiency in emotion regulation strategies and an anxious attachment style can predict psychopathological symptoms and adaptive problems. More generally, difficulties in emotional regulation and an insecure attachment style are associated with psychological problems, being related to various neuropsychiatric disorders, such as depression (Arditte and Joormann, 2011; Zheng et al., 2020), bipolar affective disorder (Wagner-Skacel et al., 2020), anxiety (Cassidy et al., 2009; Read et al., 2018), borderline personality disorder (Agrawal et al., 2004; Daros and Williams, 2019), eating disorders (McLean et al., 2007; Tasca, 2019), and stress (Moore et al., 2008). Therefore, understanding the underlying mechanisms of these factors is crucial for improving mental health interventions and outcomes.

Extensive research establishing a relationship between emotion regulation and attachment orientations has provided valuable evidence (Girme et al., 2021). In a study conducted by Guzmán-González et al. (2016), a relationship between difficulties in emotion regulation and different attachment styles was found, using the Close Relationship Experiences Questionnaire (ECR-E) (Brennan et al., 1998), which evaluates the level of security in attachment, and the Difficulties in Emotional Regulation Scale (DERS-E) (Gratz and Roemer, 2004). In this study, they showed that people with secure attachment present fewer emotion regulation difficulties while subjects with insecure attachment exhibit higher emotion regulation difficulties. In particular, people with anxious attachment present more impulse control difficulties, while people with avoidant attachment present higher emotional confusion and lack of emotional awareness due to having difficulties in identifying their emotions (Guzmán-González et al., 2016). Despite the contributions of the research by Guzmán-González et al. (2016), there are few experimental studies on emotional regulation and attachment styles. Experimental models may provide valuable information for basic and clinical research.

Different attachment orientations have shown preferences for using different emotional regulation strategies. Vrtička et al. (2012) found that individuals with secure attachment tend to use cognitive reappraisal whenever they face situations that can be socially threatening. Cognitive reappraisal is an antecedent-focused emotion regulation strategy that involves changing the way one’s think about a stressful situation in order to reduce its emotional impact. This might involve reframing the situation in a more positive way, focusing on the potential benefits or opportunities that presents (Gross, 2002). In contrast, individuals with an avoidant attachment orientation tend to use deactivation strategies by reducing the activation of the attachment system, since they have difficulties using cognitive reappraisal when experience unpleasant social emotions (Mikulincer and Shaver, 2007). Therefore, individuals with avoidant attachment use predominantly behavioral inhibition or expressive suppression of emotions which is known as response-focused emotion regulation (Gross, 2002). Furthermore, individuals with an anxious attachment orientation tend to use hyperactivation strategies, increasing the intensity of negative social situations, contrasting with the decreased emotional response presented in people with secure attachment. This type of response is related to hypervigilance for cues of rejection or social support (Mikulincer and Shaver, 2019).

Moreover, adult attachment is a broad concept that encompasses both interpersonal and non-interpersonal aspects. While attachment is typically thought of as being related to close relationships with others, it can also be relevant in non-interpersonal contexts. For example, someone with a secure attachment orientation may be better able to regulate their emotions when faced with a challenging task or situation, even if they are not interacting with another person (Mikulincer and Shaver, 2007). Slatcher and Pennebaker (2006) used a non-interpersonal task (expressive writing) to examine the relationship between attachment and emotional expression. The authors found that individuals with a secure attachment orientation were more likely to express a wider range of emotions in their writing, suggesting that they were more able to regulate their emotions in a non-interpersonal context. Similarly, a study by Rholes et al. (1999) found that individuals with a secure attachment orientation were better able to regulate their emotions in response to a stressful task, while those with an insecure attachment orientation were more likely to experience negative emotions and greater physiological arousal. Overall, these findings suggest that attachment orientations can influence non-interpersonal emotion regulation (Rholes et al., 1999; Slatcher and Pennebaker, 2006; Mikulincer and Shaver, 2007). However, how the different emotional aspects of non-interpersonal emotion regulation are related to the two dimensions of adult attachment in Latin American samples are poorly understood. In particular, the regulation of valence, arousal, and dominance using strategies such as expressive suppression and cognitive reappraisal.

In the present study, we use a classic non-interpersonal emotion regulation task in which participants have to rate different dimensions of the emotional experience such as valence, which refers to how pleasant or unpleasant an event is perceived; arousal, which refers to how intense the emotion is perceived and, finally, dominance, which has been less studied and is the degree of control over the emotion experienced. The inclusion of the assessment of these three dimensions is relevant since according to Silva (2011) they are related to the active organization of the body in the face of a stimulus that generates an emotional response and, therefore, they contribute to the adaptive process of the human being. By including these three variables, it is possible to study in greater detail the differences at the level of emotional regulation. In our behavioral paradigm all participants were instructed to actively attend, reappraise, or suppress their emotions while emotional neutral or negative images were presented. Then, participants rated their emotional experience. The level of success in the implementation of emotion regulation strategies was measured by the score variations in the valence, arousal, and dominance of emotions.

We hypothesize that different attachment orientations present different types of associations with the effectiveness of the implementation of the emotional regulation strategies, both in the implementation of cognitive reappraisal and in expressive suppression during an emotion regulation task. We attempt to answer the question of whether there is a relationship between the implementation of emotional regulation strategies and the dimensions of anxiety and/or avoidance in attachment. Therefore, our research aimed to determine whether there is an association between the implementation of emotional regulation strategies with the dimensions of anxiety and/or avoidance attachment under the two-dimensional model of adult attachment (Fraley and Waller, 1998).

Our research may have important implications for our understanding of the relationship between attachment orientations and emotional regulation, as well as for the development of interventions to help individuals with insecure attachment regulate their emotions more effectively. By providing new insights into the challenges faced by individuals with different attachment orientations, this research can contribute with valuable information for the development of targeted therapeutic approaches that can help these individuals improve their emotional regulation skills.

## 2. Materials and methods

This study was not preregistered. We report how we determined our sample size, all data exclusions, all manipulations, and all measures in the study. This study was conducted between March and October 2022.

### 2.1. Participants

Data was collected from forty-four Chilean Latin-American adults (28 females) between 18 and 50 years old [mean age = 26.91; standard deviation (SD) = 8.81]. The sample size was calculated using G*Power 3.1.9.7 software^1^ considering an effect size of 0.52, alpha value of 0.05, and a power of 0.95 (Faul et al., 2007). All participants had a normal or corrected-to-normal vision and reported not suffering from neurological or psychiatric conditions. The Scientific Ethics Committee of the Universidad Católica del Norte approved procedures, and all participants signed an informed consent form before the beginning of the study (research project number 099/2021). We have complied with APA ethical standards in the treatment of our sample.

### 2.2. Instruments

#### 2.2.1. Experiences in close relationships questionnaire ECR-12

The Experiences in Close Relationships questionnaire (ECR-12) developed by Brennan et al. (1998) aims to assess adult attachment based on the evaluation of two dimensions: anxiety and avoidance. In this study, we used the validated Chilean version (Guzmán-González et al., 2020), which is a short version in Spanish comprised of 12 items. It has two subscales of 6 items each: attachment avoidance (e.g., “*I get nervous when my partner becomes too emotionally intimate with me*”) and attachment anxiety (e.g., “*I need my partner to constantly reassure me that their love me*”). These items are answered using a seven-point Likert format, ranging from (1) totally disagree to (7) totally agree. High scores on a subscale reflects a higher level of anxiety attachment and/or avoidance attachment, respectively. The Chilean ECR-12 (Guzmán-González et al., 2020) preserved the good psychometric properties of the 36-item Chilean ECR version (Spencer et al., 2013) as well as of the original ECR (Brennan et al., 1998).

### 2.3. Emotion regulation experimental paradigm

To assess the effectiveness of the implementation of the emotional regulation strategies, an experimental emotional regulation task adapted from Ochsner et al. (2004), Vrtička et al. (2012), and Schlumpf et al. (2019), was used which was programmed using Presentation Software^®^ by Neurobehavioral Systems (Version 18.0, Neurobehavioral Systems, Inc., Albany, CA, USA).^2^ The set of stimuli consisted of images taken from the International Affective Picture System (IAPS) (Lang et al., 2005), which has shown adequate psychometric properties in the Chilean context in sets 7 and 14 (Silva, 2011). A total of 60 images were chosen, including 45 emotional negative and 15 emotional neutral which were presented under 3 experimental conditions: “*Natural*,” “*Reappraise*,” and “*Suppress*.”

Participants had first a training session to ensure they got familiar with the experiment setup and the goal of each trial condition which consisted of 3 blocks and 3 trials for each condition. Participants were instructed to score different dimensions of their emotions while observing emotional o neutral pictures. Specifically, the experimenter instructed the participants using visual aid before starting the experimental task “*A picture image will be presented on this monitor screen that can evoke negative emotions or they can be neutral images. Before the image appears, 3 possible instructions will be presented: ‘Natural’, ‘Reappraise’, or ‘Suppress*’.” Firstly, when the “*Natural*” instruction cue appeared on the screen, subjects had to actively observe the picture paying attention to their emotion experienced. In order to engage with the picture seen, they were instructed to think they were part of the observed situation in the picture. Secondly, when the “*Reappraise*” instruction cue appeared, participants had to look at the picture and try to reduce the emotional impact by implementing cognitive reappraisal, for example, imagining that the scene was part of a movie with a montage of actors and makeup, or the scenario presented on the image would later conclude in a happy ending. This way, participants were trained in self- or situation-based cognitive reappraisal strategies (Ochsner et al., 2004). The self-based reappraisal strategies consisted of (a) observing the images in the third person, that is, without emotional involvement, and (b) imagining that the images are fictitious or not representative of a real event, while the situation-based reappraisal strategy consisted of imagining an improvement in the observed situation. Finally, when the “*Suppress*” instruction cue appeared, participants were instructed to look at the image and regulate the emotion evoked avoiding expressing the emotional behavior or not allow themselves to feel the evoked emotions.

After observing each image following the respective instruction (“*Natural*,” “*Reappraise*,” or “*Suppress*”), the participants rated the emotion experienced through a 1 to 7 Likert scale based on the Self-Assessment Manikin (Bradley and Lang, 1994) according to valence (1 = unpleasant, 7 = very pleasant), arousal (1 = not arousing, 7 = highly arousing), and dominance (1 = low control, 7 = high control). At the end of the practice, participants were asked the strategies they used to control compliance with the instructions.

**FIGURE 1:**
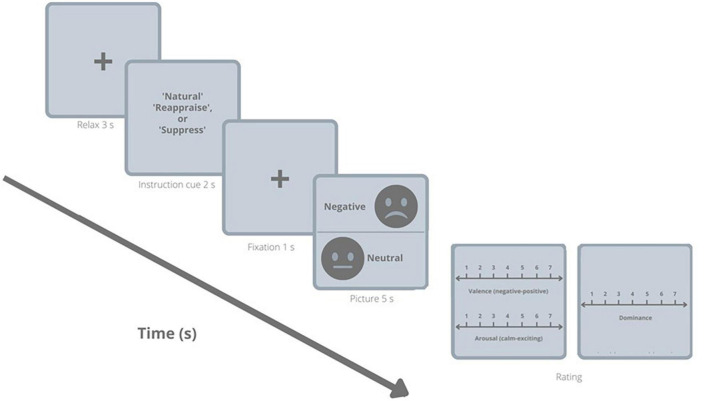
The emotion regulation task. Participants were asked to actively look at neutral or negative pictures (“*Natural*” condition) or to regulate their upcoming emotional response toward negative pictures using two different emotion regulation strategies (cognitive reappraisal or expressive suppression). Ratings of the subjective valence, the arousal, and the dominance were assessed directly after each picture presentation.

The experimental session consisted of a randomized 12 blocks presentation of 5 pictures for each of the three conditions (“*Natural*,” “*Reappraise*,” and “*Suppress*”). Specifically, for the “*Natural*” condition a total of 30 trials were presented and included: 15 negative and 15 neutral pictures. This way the “*Natural*” condition can be divided afterward into a natural neutral condition and a natural negative condition: “*Natural-neu*” and “*Natural-neg*”, respectively. For the “*Reappraise*” and the “*Suppress*” condition 15 trials, respectively, were presented and included negative pictures only. For the three conditions, each block followed a specific sequence as shown in the Figure 1 and includes: (1) A gray background with a fixation cross “ + ” appeared during 3 s in the middle of the screen and served to focus the participant’s attention; (2) The task instruction indicating the condition block “*Natural*,” “*Reappraise*,” or “*Suppress*” was presented during 2 s; (3) A cross fixation appeared again for 1 s in the middle of the screen. (4) The picture was shown (negative or neutral) for 5 s. (5) Finally, the Likert scales screen allowing to manually (with a computer mouse) rate the valence, the arousal, and the dominance of the emotion experienced was presented (Figure 1). This was repeated until all blocks were completed. At the end of each block, a pause was displayed on the monitor before continuing to the next.

### 2.4. Calibration of IAPS picture sets

A set of images from the International Affective Picture System (IAPS) bank (Lang et al., 2005) was chosen for each of the experimental conditions. The pictures for the “*Natural*,” “*Reappraise*,” and “*Suppression*” were selected considering previous research using this emotion regulation experimental paradigm (Moser et al., 2009; Schlumpf et al., 2019), and the valence, the arousal, and the dominance reported in the IAPS study (Lang et al., 2005). Supplementary Table 1 shows the 15 images chosen for each condition (Supplementary Table 1). “*Natural*” condition was divided into “*Natural-neg*” condition which contained a set of 15 negative pictures, and “Natural-neu” condition containing a set of 15 neutral pictures. The Supplementary Table 2 shows the descriptive statistics of the images selected by valence, arousal, and dominance per condition (Supplementary Table 2).

In order to verify that the selection of IAPS pictures chosen for each conditions was equivalent in terms of valence, arousal, and dominance for the “*Natural-neg*,” “*Suppress*,” and “*Reappraise*” conditions; and these, in turn, were different from the “Natural-neu” condition, we performed a parametric (1-way ANOVA) and/or non-parametric (Kruskal-Wallis) hypothesis tests depending on the distribution of the data previously evaluated using the normality test Shapiro-Wilk (Supplementary Table 3).

A non-parametric Kruskal-Wallis’s test was performed, and we found significant differences for valence between conditions (*H* = 35.43; *p* < 0.0001). Additionally, Dunn’s non-parametric test of multiple comparisons was implemented (considering an Alpha of 0.05), and as expected, significant differences were observed between the valence values of the “*Natural-neu*” condition picture set compared to the “*Natural-neg*” (*d* = 6.2; *p* < 0.0001), “*Suppress*” (*d* = 5.73; *p* < 0.0001) and “*Reappraise*” (*d* = 4.97; *p* = 0.0007) conditions picture sets but there were no significant differences between the sets of negative pictures of each condition (Supplementary Table 4). Subsequently, a 1-way ANOVA parametric test was applied, and we found significant differences for the arousal between the conditions (*F* = 46.66; *p* < 0.0001). Then, Tukey’s test of multiple comparisons was implemented (Alpha = 0.05), and as expected, significant differences were observed between the arousal values of the “*Natural-neu*” condition with the “*Natural-neg*” (*d* = −4.86; *p* < 0.0001; 95% CI [1.94, 3.27]), the “*Suppress*” (*d* = −3.43; *p* < 0.0001; 95% CI [1.62, 2.93]), and the “*Reappraise*” (*d* = −3.53; *p* < 0.0001; 95% CI [1.68, 3.01]) conditions, but no significant differences were observed between these three conditions (Supplementary Table 5). The same previous procedure was carried out to analyze the dominance values and we found significant differences between the conditions (*F* = 54.02; *p* < 0.0001). According to the Tukey’s test, the differences between conditions were also found between the “*Natural-neu*” condition with the “*Natural-neg*” (*d* = 4.14; *p* < 0.0001; 95% CI [−2.85, −1.70]), “*Suppress*” (*d* = 5.02; *p* < 0.0001; 95% CI [−2.95, −1.80]), and “*Reappraise*” (*d* = 3.71; *p* < 0.0001; 95% CI [−2.67, −1.52]) conditions, but not between the latter three (Supplementary Table 6).

**FIGURE 2:**
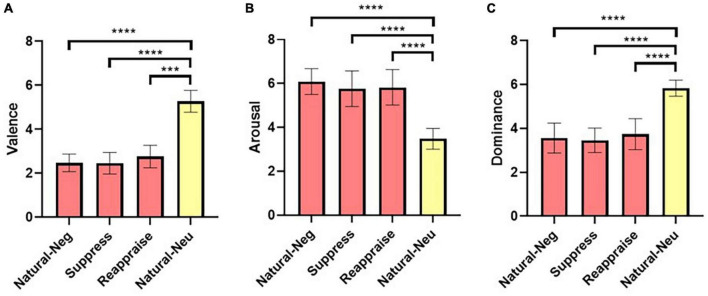
Calibration of selected IAPS pictures. Figure shows the valence, arousal, and dominance values of the selected IAPS pictures used for each condition of the emotion regulation task. No significant differences were observed between the pictures selected for valence **(A)**, arousal **(B)**, and dominance **(C)** for the “*Natural-neg*,” “*Suppress*,” and “*Reappraise*” conditions, but all of them were significantly different when compared to the pictures selected for the “Natural-neu” condition. Statistical analyzes were performed using Kruskal-Wallis **(A)** and 1-way ANOVA **(B,C)**, and multiple comparison tests using Dunn’s **(A)** and Tukey’s **(B,C)** test. Natural-neg: natural condition containing negative valence pictures; Natural-neu: natural condition containing neutral valence pictures. ^***^p < 0.001; ^****^p < 0.0001.

In summary, the pictures selected for the emotion regulation task were adequate, since there were no differences between them, in terms of valence, arousal, and dominance for the conditions in which participants were required to engage and evoke negative emotions naturally (“*Natural-neg*”), or as they attempted to suppress their emotional expression (“*Suppression*”), or when they re-evaluate the meaning of the pictures (“*Reappraise*”). Additionally, for the condition in which it was required to present neutral pictures to the participants (“*Natural-neu*”), the set of pictures was significantly different from the other conditions for the values of valence, arousal, and dominance, being less unpleasant, less intense, and evoked greater emotional control than the negative pictures from the other conditions (Figure 2). In conclusion, the use of the set of selected pictures for each condition were appropriate to use in our emotional regulation paradigm.

### 2.5. Data analyses

All data were analyzed using GraphPad Prism version 8 for Windows (GraphPad Software, La Jolla CA, USA).^3^ Traditional descriptive statistics were used. The Shapiro-Wilk normality distribution test was performed to determine the use of parametric or non-parametric statistical hypothesis tests (Mishra et al., 2019). To analyze the differences between emotion regulation conditions ANOVA or Friedman tests were performed, as appropriate, and *post-hoc* tests with corrections for multiple comparisons were used. Levels of anxiety and avoidance attachment were compared using a Wilcoxon signed-rank test. Size effect was calculated using Cohen’s d values. Additionally, correlation analyzes were performed using Spearman’s coefficient to determine the degree of association between the values of emotional regulation strategies (reappraisal and suppression) and the dimensions of anxiety and avoidance levels of attachment.

## 3. Results

### 3.1. Emotion regulation task performance

The mean, standard deviation, and standard error of the mean for the values of valence, arousal, and dominance in the emotional regulation task per condition were calculated for all participants (Supplementary Table 7) and a normality distribution test (Supplementary Table 8).

**FIGURE 3:**
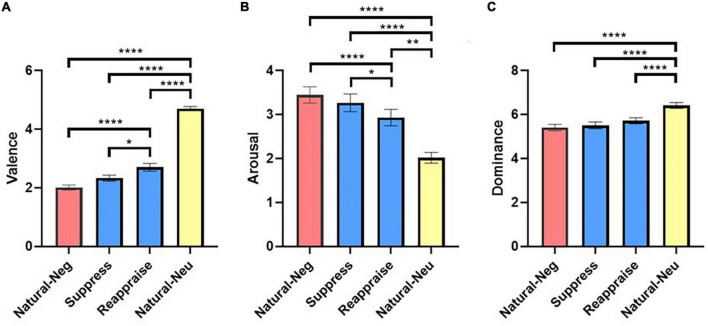
Performance on the emotion regulation task. Figure shows significant differences between the ratings in valence **(A)**, arousal **(B)**, and Dominance **(C)** when comparing “*Natural-neu*” to the rest of conditions for all participants. Ratings of valence and arousal in “*Natural-neg*” were significantly different when compared to “*Reappraise*” but not when compared to “*Suppress*,” showing that cognitive reappraisal strategy was more effective than expressive suppression to modulate emotions. *p < 0.05; ^**^p < 0.01; ^****^p < 0.0001.

Afterward, a Friedman test was performed to assess the variance of valence (*Q* = 106.2; *p* < 0.0001), arousal (*Q* = 71.97; *p* < 0.0001), and dominance (*Q* = 71.70; *p* < 0.0001), between the different conditions. We found differences in the three ratings values and conditions. Additionally, Dunn’s multiple comparison tests were performed to compare the rating values of valence, arousal, and dominance of the different conditions (Supplementary Tables 9–11 and Figure 3).

Specifically, we found pairwise *post-hoc* Dunn test was significant in the valence ratings between the “*Natural-Neg*” and the “*Reappraise*” (*d* = −0.92; *p* < 0.0001) conditions, the “*Natural-Neg*” and the “*Natural-Neu*” (*d* = −5.08; *p* < 0.0001) conditions, the “Suppress” and the “Reappraise” (*d* = −0.47; *p* = 0.0118) conditions, the “*Suppress*” and the “*Natural-Neu*” (*d* = −4.05; *p* < 0.0001) conditions, and the “*Reappraise*” vs. the “*Natural-Neu*” (*d* = −2.78; *p* = 0.0002) conditions (Supplementary Table 9 and Figure 3A). Additionally, we found significant differences when comparing the arousal ratings between the “*Natural-Neg*” and the “*Reappraise*” (*d* = 0.42; *p* = 0.0003) conditions, the “*Natural-Neg*” and the “*Natural-Neu*” (*d* = 1.38; *p* < 0.0001) conditions, the “Suppress” and the “*Reappraise*” (*d* = 0.26; *p* = 0.0017) conditions, the “*Suppress*” and the “*Natural-Neu*” (*d* = 1.12; *p* < 0.0001) conditions, the “*Reappraise*” and the “*Natural-Neu*” (*d* = 0.87; *p* = 0.0066) conditions (Supplementary Table 10 and Figure 3B). Finally, we found significant differences when we compared the dominance ratings between the “*Natural-Neg*” and the “*Natural-Neu*” (*d* = −1.07; *p* < 0.0001) conditions, the “*Suppress*” and the “*Natural-Neu*” (*d* = −0.96; *p* < 0.0001) conditions, and the “*Reappraise*” and the “*Natural-Neu*” (*d* = −0.77; *p* < 0.0001) conditions (Supplementary Table 11 and Figure 3C).

According to the participants performance, our emotion regulation task was optimal for studying the attachment orientations, since in the different conditions, participants showed adequate implementation of emotion regulation strategy when face emotionally charged pictures with negative content.

### 3.2. Relationship between the effectiveness of the implementation of the emotional regulation strategy and attachment dimensions

Table 1 summarizes the descriptive statistics results of the scores obtained using the ECR-12.

**TABLE 1:** Descriptive statistics of the ECR-12 score.

Participants	*n*	Age		Anxiety		Avoidance	
		Mean	SD	Mean	SD	Mean	SD
Female	28	26.79	8.51	4.22	1.79	2.36	1.39
Male	16	27.13	9.58	2.84	1.18	2.12	0.78
Total	44	26.91	8.81	3.72	1.72	2.27	1.21

SD, standard deviation.

**FIGURE 4:**
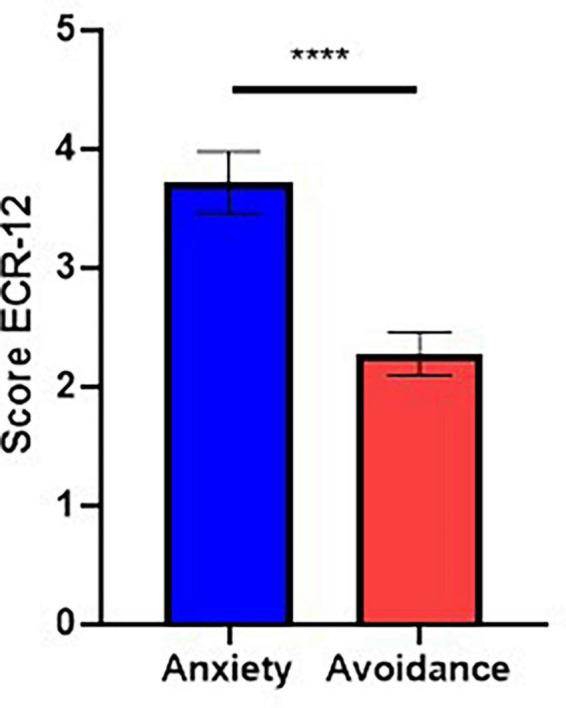
Scores obtained using ECR-12. Participants showed higher levels of anxiety attachment than avoidance attachment (*d* = 0.98; *W* = –732; *p* < 0.0001; 95% CI [–2.00, –0.67]). ^****^p < 0.0001.

We applied a Wilcoxon signed-rank test (*d* = 0.98; *W* = −732; *p* < 0.0001, 95% CI [−2.00, −0.67]) and observed that in our sample the participants presented higher levels of anxiety attachment compared to avoidance attachment (Supplementary Table 12 and Figure 4).

**FIGURE 5:**
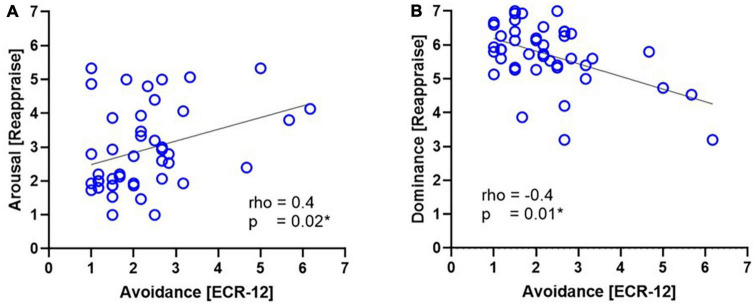
Correlations between avoidance attachment levels (ECR-12) and emotion regulation task ratings during reappraisal. The figure shows the correlations between values of the avoidance dimension score using the ECR-12 and the arousal score **(A)** and dominance **(B)** during cognitive reappraisal in the emotion regulation task.

To determine if there was a relationship between emotion regulation effectiveness and attachment orientations, we performed a Spearman rho’s correlation test and found an association between arousal and dominance values during cognitive reappraisal with levels of avoidance attachment. Specifically, we found a positive correlation in which, the greater the emotional intensity during the reappraisal, the higher the scores in the avoidance dimension (*Rho* = 0.4; *p* = 0.02; 95% CI [0.05, 0.59]; Figure 5A). Additionally, we found a negative correlation where, at a lower level of emotional control (dominance) during cognitive reappraisal, there were higher scores in the avoidance dimension of attachment (*Rho* = −0.4; *p* = 0.01; 95% CI [−0.61, −0.07]; Figure 5B; Supplementary Table 13).

Likewise, we found a negative correlation between the dominance values during emotional suppression with the avoidance dimension of attachment, in which the higher the score in the avoidance dimension, the lower the emotional control (*Rho* = −0.3; *p* = 0.04; 95% CI [−0.57, −0.02]; Supplementary Figure 1).

No other correlations were found between attachment dimensions and emotional ratings during “*Reappraise*” (Supplementary Tables 13, 14) or “*Suppress*” (Supplementary Tables 15, 16).

## 4. Discussion

The aim of this study was to determine the level of association between the effectiveness of the implementation of emotional regulation strategies and the dimensions of anxiety and avoidance attachment orientations.

In attachment research, two traditions can be distinguished: one originating from evolutionary psychology and the other from social psychology. In the former tradition, the focus is on the mental state regarding the relationship with parental figures, while in the latter perspective, the functioning in current relationships is evaluated. Both traditions have tended to privilege different ways of evaluating and coding attachment. In the first case, narrative interviews are used to distinguish attachment styles, while in the second tradition, self-report questionnaires are used to distinguish variations in two dimensions or orientations, anxiety and avoidance in attachment (Del Giudice and Belsky, 2010; Van IJzendoorn and Bakermans-Kranenburg, 2010; Cassidy and Shaver, 2016; Mikulincer and Shaver, 2016). Our study follows the second tradition, which is why we use the concept of attachment dimensions or orientations.

Based on our results obtained through the administration of the ECR-12 instrument and the emotion regulation task, a relationship was shown between attachment avoidance and the implementation of cognitive reappraisal as an emotional regulation strategy. Interestingly, we found that during cognitive reappraisal implementation, as avoidance attachment levels increase, arousal levels increase as well, indicating a propensity in participants with avoidance tendencies to have difficulties in reducing arousal when implementing the cognitive reappraisal strategy in our emotion regulation task. In the same line, a similar pattern of association was shown for dominance, whereas avoidance levels increase, the level of control of emotions decreases, indicating a lower perception of emotion dominance during cognitive reappraisal strategy implementation. Both results are consistent with our hypothesis that individuals with more avoidance in attachment levels exhibit a less efficient cognitive reappraisal performance, in which they struggle reducing their emotional intensity and controlling their emotions.

Several research have reported that people with higher attachment avoidance tend to regulate their emotions through suppression or inhibition, since they tend to deactivate their attachment system (Garrido-Rojas, 2006; Mikulincer and Shaver, 2016; Long et al., 2020). However, in our study we did not find a relationship between the attachment avoidance dimension and regulation parameters during suppression. We interpret this finding is in line with evidence showing that suppression strategy had no effect on self-report of affective experience (Gross, 2001; Goldin et al., 2008). In fact, according to the latest research on emotional regulation strategies, emotional suppression can reduce the observable or expressive aspects of emotions as well as certain peripheral physiological responses, but it doesn’t have an effect on diminishing the related emotional experience (Mohammed et al., 2021). Considering that expressive suppression, as a response-focused regulation strategy, target the expressive consequences of emotion, thus it is not possible to evaluate its effectiveness in regulate the expressive component of emotions via self-report as we have done in our study design. According to a meta-analysis conducted by Webb et al. (2012), which investigated the efficacy of emotion regulation strategies based on the emotion-process model, it was found that inhibiting the outward expression of emotions was effective. However, attempting to suppress the experience of emotions or thoughts related to the emotional stimulus event was found to be ineffective. One limitation of our study is the use of self-report as a measure that only allow us to access to the experience of the emotional process. The current method of measuring the effectiveness of emotion regulation only enables the evaluation of the impact of reappraisal on one’s experience, rather than directly assessing the effect of suppression on outward expression. Further research is needed in order to incorporate an assessment of the expressive outcomes of emotions or the physiological aspects that are successfully regulated (or not), rather than relying solely on self-reported emotional experiences. For instance, Trentini and Dan-Glauser (2023) hypothesized that conflicting findings on the effectiveness of emotion regulation strategies (reappraisal and suppression) could be due to limitations in temporal resolution and proposed a difference index to increase sensitivity to signal differences which may be a useful tool for measuring emotion regulation effectiveness.

Efficacy in implementing reappraisal helps to increase dominance (Gross and John, 2003), therefore, people who are inclined to avoidance in attachment, having difficulties implementing reappraisal, present less dominance of emotions. On the other hand, the effectiveness in the implementation of cognitive reappraisal is associated with less activity in the brain areas associated with emotional activation (Goldin et al., 2008), therefore, in the case of our results where the participants with higher avoidance levels of attachment presented an increase in arousal when implementing cognitive reappraisal, they could present difficulties in reducing the levels of brain activation associated with emotional reactivity.

There are several reasons why people with a tendency to avoidance in attachment present difficulties in implementing cognitive reappraisal. Firstly, avoidant people tend to repress or avoid their emotions, instead of trying to regulate them, thus it can be more difficult to implement reappraisal, since it requires knowing and accepting one’s own emotions. Secondly, these people could present more difficulties in identifying and expressing emotions, making it difficult to implement cognitive reappraisal Thirdly, they would have a limited repertoire of regulation strategies, without having developed the necessary resources to use cognitive reappraisal effectively. Fourthly, they may have negative beliefs or attitudes regarding emotions, which may make it difficult for them to use emotional regulation strategies (Fraley and Shaver, 1998; Shaver and Mikulincer, 2002; Mikulincer and Shaver, 2007).

In this line, our results are consistent with what was found in Guzmán-González et al. (2016), where they evaluated the relationship between attachment styles and emotional regulation, using the Experiences in Close Relationships (ECR) scales and the Difficulties in Emotional Regulation (DERS) questionnaire. These authors found that the secure attachment style is associated with fewer emotional regulation difficulties in all its dimensions. Additionally, people who had high levels of avoidance, with avoidant and fearful attachment, presented greater difficulties in inattention to emotions and emotional confusion, that is, difficulties in attending to and recognizing their emotions (Guzmán-González et al., 2016).

Furthermore, is noteworthy that the results shown using our experimental paradigm confirms what is stated in the bibliographical background that postulates that cognitive reappraisal is a more successful strategy to regulate activation at the physiological and subjective level of the emotional experience (Gross, 1998). Moreover, it has been shown that the positive effects of cognitive reappraisal are also much more durable than other strategies (Ochsner and Gross, 2005). Therefore, our version of the emotion regulation paradigm is useful for future research. One future direction is using this experimental design coupled to neuroimaging methods such as fMRI (Ochsner et al., 2002), EEG (Ertl et al., 2013; Schlumpf et al., 2019), or eye-tracker systems (Bradley et al., 2008), which might allow provide answers about how brain dynamics and pupil dynamics change induced by emotional regulation as a function of attachment and their relationship with behavioral parameters of the emotional experience. On the other hand, a study can be carried out from a categorical perspective of the attachment theory, in which people could be classified according to their predominant attachment style (Bartholomew, 1990) and, accordingly, evaluate the effects that may exist in the different emotion regulation conditions.

Besides, one important aspect of our sample is that, based on the results of the ECR-12 instrument, the participants showed higher levels of anxiety than avoidance attachment, which is consistent with studies conducted in the general population (Chopik and Edelstein, 2014; Karataş et al., 2017; Guzmán-González et al., 2020). Although these results may reflect cultural factors, there are few studies that evaluate the distribution of attachment dimensions in Latin America. Studies that evaluate attachment in various cultures show that in those societies that are less individualistic, anxiety in relationships (Wang and Mallinckrodt, 2006) is greater than in westernized countries. This could be applied to the Latin American context; however, more conclusive studies are required. Regarding Chile, it is possible to consider the results obtained by Garrido et al. (2009), where they evaluated the distribution of adult attachment in Chile using the CaMir instrument, obtaining that, among insecure attachments, anxious (preoccupied) attachment is predominant.

Based on the results of our research, we conclude that the attachment dimensions, particularly the avoidance attachment dimension, influence the effectiveness of the implementation of cognitive reappraisal as an emotional regulation strategy, which is reflected in the relationship between attachment avoidance and parameters of emotional regulation. Specifically, the difficulties in reducing arousal and the decrease in dominance during cognitive reappraisal implementation.

## Data availability statement

The raw data supporting the conclusions of this article will be made available by the authors, without undue reservation.

## Ethics statement

The studies involving human participants were reviewed and approved by Comité de Ética Científica de la Universidad Católica del Norte. The patients/participants provided their written informed consent to participate in this study.

## Author contributions

MD-S: conceptualization, data curation, formal analysis, funding acquisition, investigation, methodology, software, validation, project administration, resources, supervision, visualization, writing—original draft, and writing—review and editing. MG-G: conceptualization, validation, methodology, supervision, and writing—review and editing. JB: conceptualization, investigation, methodology, supervision, and writing—original draft. CatC, CF-G, CF-V, and JS: conceptualization, investigation, methodology, and writing—original draft. OV-G: conceptualization, supervision, and writing—review and editing. CarC: methodology and writing—review and editing. AS-C: data curation, methodology, software, and validation. MP-B: writing—review and editing and supervision. JM-M: conceptualization, validation, supervision, visualization, and writing—review and editing. All authors contributed to the article and approved the submitted version.

## Footnotes

1
http://www.gpower.hhu.de/

2
www.neurobs.com

3
www.graphpad.com
