# Supplementary material for: Emotion regulation strategies and the two-dimensional model of adult attachment: a pilot study
Source: Front Behav Neurosci. 2023 Jul 7;17:1141607. doi: 10.3389/fnbeh.2023.1141607 (PMC10359990; doi:10.3389/fnbeh.2023.1141607)
Supplement: Supplementary file 1 [file Data_Sheet_1.docx]

**Supplementary Material**

- 1. **Supplementary Tables**

**Table S1. Selected IAPS pictures per condition (Lang et al., 2005)**

|  | Experimental Conditions | | | |
| --- | --- | --- | --- | --- |
| N° | Natural-neg | Suppress | Reappraise | Natural-neu |
| 1 | 2683 | 2095 | 2130 | 7009 |
| 2 | 2703 | 2205 | 2700 | 7011 |
| 3 | 2710 | 2900 | 2751 | 7012 |
| 4 | 2717 | 6211 | 2811 | 7018 |
| 5 | 3220 | 6231 | 3550 | 7042 |
| 6 | 3230 | 6250 | 6260 | 7045 |
| 7 | 3500 | 6312 | 6350 | 7061 |
| 8 | 6300 | 6560 | 6530 | 2190 |
| 9 | 6315 | 6563 | 6561 | 2215 |
| 10 | 6360 | 6838 | 6562 | 2359 |
| 11 | 6540 | 9041 | 6825 | 2499 |
| 12 | 6550 | 9250 | 6834 | 2518 |
| 13 | 6840 | 9421 | 9050 | 2521 |
| 14 | 9429 | 9427 | 9419 | 2593 |
| 15 | 9900 | 9435 | 9425 | 2594 |

Natural-neg: Natural condition containing negative valence pictures; Natural-neu: Natural condition containing neutral valence pictures.

**Table S2. Descriptive statistics of the selected IAPS pictures per condition.**

|  | Valence | | Arousal | | Dominance | |
| --- | --- | --- | --- | --- | --- | --- |
|  | Mean | SD | Mean | SD | Mean | SD |
| Natural-neg | 2.48 | 0.40 | 6.09 | 0.59 | 3.56 | 0.68 |
| Suppress | 2.50 | 0.50 | 5.76 | 0.81 | 3.46 | 0.56 |
| Reappraise | 2.77 | 0.51 | 5.83 | 0.81 | 3.74 | 0.71 |
| Natural-neu | 5.30 | 0.50 | 3.48 | 0.48 | 5.83 | 0.37 |

Natural-neg: Natural condition containing negative valence pictures; Natural-neu: Natural condition containing neutral valence pictures. SD = Standard Deviation.

Table S3 shows the results of the normality tests applied to the selected IAPS pictures for each variable of the conditions, using the Shapiro-Wilk test showing that the valence of the pictures selected for the condition Natural-neg do not pass the normality test.

**Table S3. Normality test of the IAPS pictures values selected per condition.**

|  | Valence | | Arousal | | Dominance | |
| --- | --- | --- | --- | --- | --- | --- |
|  | W | p-value | W | p-value | W | p-value |
| Natural-neg | 0.86 | **0.03*** | 0.92 | 0.17 | 0.97 | 0.85 |
| Suppress | 0.95 | 0.50 | 0.91 | 0.15 | 0.93 | 0.24 |
| Reappraise | 0.94 | 0.33 | 0.92 | 0.19 | 0.97 | 0.78 |
| Natural-neu | 0.94 | 0.33 | 0.94 | 0.34 | 0.96 | 0.63 |

Natural-neg: Natural condition containing negative valence pictures; Natural-neu: Natural condition containing neutral valence pictures. W: Shapiro-Wilk Statistics.

**Table S4.** **Multiple comparison test for the valence of IAPS pictures between conditions**

| Dunn’s test | Mean rank diff. | Summary | Adjusted p-value |
| --- | --- | --- | --- |
| Natural-Neg vs. Suppress | 1.067 | ns | >0.9999 |
| Natural-Neg vs. Reappraise | -7.667 | ns | >0.9999 |
| Natural-Neg vs. Natural-Neu | -32.20 | **** | **<0.0001** |
| Suppress vs. Reappraise | -8.733 | ns | >0.9999 |
| Suppress vs. Natural-Neu | -33.27 | **** | **<0.0001** |
| Reappraise vs. Natural-Neu | -24.53 | *** | **0.0007** |

Natural-neg: Natural condition containing negative valence pictures; Natural-neu: Natural condition containing neutral valence pictures.; ns: not significant.

**Table S5.** **Multiple comparison test for the arousal of IAPS pictures between conditions**

| Tukey’s test | Mean Diff. | 95.00% CI of diff. | Summary | Adjusted p-value |
| --- | --- | --- | --- | --- |
| Natural-Neg vs. Suppress | 0.3240 | -0.3415 to 0.9895 | ns | 0.5736 |
| Natural-Neg vs. Reappraise | 0.2633 | -0.4022 to 0.9288 | ns | 0.7222 |
| Natural-Neg vs. Natural-Neu | 2.607 | 1.942 to 3.273 | **** | **<0.0001** |
| Suppress vs. Reappraise | -0.06067 | -0.7262 to 0.6048 | ns | 0.9950 |
| Suppress vs. Natural-Neu | 2.283 | 1.618 to 2.949 | **** | **<0.0001** |
| Reappraise vs. Natural-Neu | 2.344 | 1.679 to 3.009 | **** | **<0.0001** |

Natural-neg: Natural condition containing negative valence pictures; Natural-neu: Natural condition containing neutral valence pictures.; ns: not significant.

**Table S6. Multiple comparison test for the dominance of IAPS pictures between conditions**

| Tukey’s test | Mean Diff. | 95.00% CI of diff. | Summary | Adjusted p-value |
| --- | --- | --- | --- | --- |
| Natural-Neg vs. Suppress | 0.1000 | -0.4751 to 0.6751 | ns | 0.9673 |
| Natural-Neg vs. Reappraise | -0.1793 | -0.7544 to 0.3957 | ns | 0.8420 |
| Natural-Neg vs. Natural-Neu | -2.272 | -2.847 to -1.697 | **** | **<0.0001** |
| Suppress vs. Reappraise | -0.2793 | -0.8544 to 0.2957 | ns | 0.5755 |
| Suppress vs. Natural-Neu | -2.372 | -2.947 to -1.797 | **** | **<0.0001** |
| Reappraise vs. Natural-Neu | -2.093 | -2.668 to -1.518 | **** | **<0.0001** |

Natural-neg: Natural condition containing negative valence pictures; Natural-neu: Natural condition containing neutral valence pictures; ns: not significant.

**Table S7. Descriptive statistics of ratings for valence, arousal, and dominance of the emotional regulation task.**

|  | Valence | | | *Arousal* | | | Dominance | | |
| --- | --- | --- | --- | --- | --- | --- | --- | --- | --- |
|  | Mean | SD | SEM | Mean | SD | SEM | Mean | SD | SEM |
| Natural-neg | 2.01 | 0.57 | 0.09 | 3.45 | 1.22 | 0.18 | 5.41 | 0.99 | 0.15 |
| Suppress | 2.33 | 0.67 | 0.10 | 3.27 | 1.35 | 0.20 | 5.51 | 1.00 | 0.15 |
| Reappraise | 2.70 | 0.89 | 0.13 | 2.93 | 1.24 | 0.19 | 5.72 | 0.91 | 0.14 |
| Natural-neu | 4.70 | 0.49 | 0.07 | 2.01 | 0.82 | 0.12 | 6.42 | 0.89 | 0.13 |

Natural-neg: Natural condition containing negative valence pictures; Natural-neu: Natural condition containing neutral valence pictures. SD = Standard Deviation; SEM = Standard Error of the Mean.

The Shapiro-Wilk test was applied to evaluate the distribution of the ratings data for each condition in the emotional regulation task. We found that the valence values of the ‘Natural-neu’ condition, and the arousal values in the ‘Reappraise’ and the ‘Natural-neu’, and the dominance values in all conditions, did not pass the normality test (**Supplementary Table S8**).

**Table S8. Normality test of ratings per condition in the emotion regulation task.**

|  | Valence | | Arousal | | Dominance | |
| --- | --- | --- | --- | --- | --- | --- |
|  | W | p-value | W | p-value | W | p-value |
| Natural-neg | 0.96 | 0.15 | 0.96 | 0.20 | 0.94 | **0.02*** |
| Suppess | 0.98 | 0.68 | 0.95 | 0.08 | 0.93 | **0.007**** |
| Reappraise | 0.97 | 0.43 | 0.92 | **0.005**** | 0.92 | **0.005**** |
| Natural-neu | 0.85 | **<0.0001****** | 0.86 | **0.0001***** | 0.63 | **<0.0001****** |

Natural-neg: Natural condition containing negative valence pictures; Natural-neu: Natural condition containing neutral valence pictures. W: Shapiro-Wilk Statistics.

**Table S9.** **Multiple comparison test for valence ratings between different conditions of the emotion regulation task**

| Dunn’s test | Rank sum diff. | Summary | Adjusted p-value |
| --- | --- | --- | --- |
| Natural-Neg vs. Suppress | -29.50 | ns | 0.0891 |
| Natural-Neg vs. Reappraise | -67.00 | ******** | **<0.0001** |
| Natural-Neg vs. Natural-Neu | -117.5 | ******** | **<0.0001** |
| Suppress vs. Reappraise | -37.50 | ***** | **0.0118** |
| Suppress vs. Natural-Neu | -88.00 | ******** | **<0.0001** |
| Reappraise vs. Natural-Neu | -50.50 | ******* | **0.0002** |

Natural-neg: Natural condition containing negative valence pictures; Natural-neu: Natural condition containing neutral valence pictures; ns: not significant.

**Table S10.** **Multiple comparison test for arousal ratings between different conditions of the emotion regulation task**

| Dunn’s test | Rank sum diff. | Summary | Adjusted p-value |
| --- | --- | --- | --- |
| Natural-Neg vs. Suppress | 5.500 | ns | >0.9999 |
| Natural-Neg vs. Reappraise | 49.50 | ******* | **0.0003** |
| Natural-Neg vs. Natural-Neu | 89.00 | ******** | **<0.0001** |
| Suppress vs. Reappaise | 44.00 | ****** | **0.0017** |
| Suppress vs. Natural-Neu | 83.50 | ******** | **<0.0001** |
| Reappraise vs. Natural-Neu | 39.50 | ****** | **0.0066** |

Natural-neg: Natural condition containing negative valence pictures; Natural-neu: Natural condition containing neutral valence pictures; ns: not significant.

**Table S11. Multiple comparison test for dominance ratings between different conditions of the emotion regulation task**

| Dunn’s test | Rank sum diff. | Summary | Adjusted p-value |
| --- | --- | --- | --- |
| Natural-Neg vs. Suppress | -6.000 | ns | >0.9999 |
| Natural-Neg vs. Reappraise | -30.00 | ns | 0.0795 |
| Natural-Neg vs. Natural-Neu | -90.00 | ******** | **<0.0001** |
| Suppress vs. Reappraise | -24.00 | ns | 0.2851 |
| Suppress vs. Natural-Neu | -84.00 | ******** | **<0.0001** |
| Reappraise vs. Natural-Neu | -60.00 | ******** | **<0.0001** |

Natural-neg: Natural condition containing negative valence pictures; Natural-neu: Natural condition containing neutral valence pictures; ns: not significant.

The normality of the distribution of the data obtained with the administration of the ECR-12 instrument for each dimension (anxiety and avoidance) was evaluated (**Supplementary Table S12**).

**Tabla S12. Normality test for anxiety and avoidance attachment dimensions scores according to the ECR-12.**

|  | ECR-12 Attachment dimensions | |
| --- | --- | --- |
| Shapiro-Wilk test | Anxiety | Avoidance |
| W | 0.9413 | 0.8361 |
| P-value | **0.0264** | **<0.0001** |
| Passed normality test (alpha=0.05)? | No | No |
| P-value summary | ***** | ******** |

ns: not significant.

**Table S13.** **Correlations between cognitive reappraisal rating values and avoidance attachment scores according to the ECR-12**

| Cognitive Reappraisal and Avoidance attachment | | | | | |
| --- | --- | --- | --- | --- | --- |
| Valence | | Arousal | | Dominance | |
| Rho | p-value | Rho | p-value | Rho | p-value |
| -0.09 | 0.52 | **0.4** | **0.02*** | **-0.4** | **0.01*** |

**Table S14.** **Correlations between cognitive reappraisal rating values and anxiety attachment scores according to the ECR-12**

| Cognitive Reappraisal and Anxiety attachment | | | | | |
| --- | --- | --- | --- | --- | --- |
| Valence | | Arousal | | Dominance | |
| Rho | p-value | Rho | p-value | Rho | p-value |
| -0.09 | 0.56 | -0.11 | 0.49 | -0.13 | 0.39 |

**Table S15.** **Correlations between expressive suppression rating values and avoidance attachment scores according to the ECR-12**

| Expressive Suppression and Avoidance attachment | | | | | |
| --- | --- | --- | --- | --- | --- |
| Valence | | *Arousal* | | Dominance | |
| Rho | p-value | Rho | p-value | Rho | p-value |
| -0.07 | 0.65 | 0.3 | 0.05 | **-0.3** | **0.03*** |

**Tabla S16. Correlations between expressive suppression rating values and anxiety attachment scores according to the ECR-12**

| Expressive Suppresion and Anxiety attachment | | | | | |
| --- | --- | --- | --- | --- | --- |
| Valence | | Arousal | | Dominance | |
| Rho | p-value | Rho | p-value | Rho | p-value |
| -0.07 | 0.67 | -0.2 | 0.19 | -0.01 | 0.96 |

- 1. **Supplementary Figures**

**Figure S1. Correlation between avoidance attachment levels and dominance during expressive suppression.** The figure shows the correlation between values of the avoidance dimension score using the ECR-12 and the dominance score during expressive suppression in the emotion regulation task.
